# Spatial distribution of cannabinoid receptor type 1 (CB_1_) in normal canine central and peripheral nervous system

**DOI:** 10.1371/journal.pone.0181064

**Published:** 2017-07-10

**Authors:** Jessica Freundt-Revilla, Kristel Kegler, Wolfgang Baumgärtner, Andrea Tipold

**Affiliations:** 1 Department of Small Animal Medicine and Surgery, University of Veterinary Medicine Hannover Foundation, Hannover, Germany; 2 Center for Systems Neuroscience, Hannover, Germany; 3 Department of Pathology, University of Veterinary Medicine Hannover Foundation, Hannover, Germany; Martin Luther University, GERMANY

## Abstract

The endocannabinoid system is a regulatory pathway consisting of two main types of cannabinoid receptors (CB_1_ and CB_2_) and their endogenous ligands, the endocannabinoids. The CB_1_ receptor is highly expressed in the central and peripheral nervous systems (PNS) in mammalians and is involved in neuromodulatory functions. Since endocannabinoids were shown to be elevated in cerebrospinal fluid of epileptic dogs, knowledge about the species specific CB receptor expression in the nervous system is required. Therefore, we assessed the spatial distribution of CB_1_ receptors in the normal canine CNS and PNS. Immunohistochemistry of several regions of the brain, spinal cord and peripheral nerves from a healthy four-week-old puppy, three six-month-old dogs, and one ten-year-old dog revealed strong dot-like immunoreactivity in the neuropil of the cerebral cortex, *Cornu Ammonis* (CA) and dentate gyrus of the hippocampus, midbrain, cerebellum, medulla oblongata and grey matter of the spinal cord. Dense CB_1_ expression was found in fibres of the globus pallidus and substantia nigra surrounding immunonegative neurons. Astrocytes were constantly positive in all examined regions. CB_1_ labelled neurons and satellite cells of the dorsal root ganglia, and myelinating Schwann cells in the PNS. These results demonstrate for the first time the spatial distribution of CB_1_ receptors in the healthy canine CNS and PNS. These results can be used as a basis for further studies aiming to elucidate the physiological consequences of this particular anatomical and cellular distribution.

## Introduction

The properties for medical intervention of the plant Marijuana (*Cannabis sativa*) have been known for centuries [1, 2]. Behavioural and pharmacological effects of its most psychoactive component, Δ^9^ –tetrahydrocannabinol (THC), can be explained by the activation of receptors localized in the nervous system [3, 4] and peripheral tissues [5]. These receptors are known as cannabinoid receptors (CBs), and along with their endogenous ligands, the endocannabinoids (ECs), and the enzymes responsible for their synthesis and degradation, constitute the endocannabinoid system [6, 7]. In mammalian tissues, two main subtypes of cannabinoid receptors, the cannabinoid receptor 1 (CB_1_) and cannabinoid receptor 2 (CB_2_), which are G protein-coupled receptors, have been recognized [5, 8] and are responsible for the transduction of different effects of ECs [9]. Furthermore, CB_1_ receptors have been shown to be primarily expressed in the central nervous system (CNS) and peripheral nervous system (PNS) while CB_2_ receptors are mostly found in cells of the immune system [10, 11].

Besides the therapeutical effects of several cannabinoids as antiemetics, analgesics, antispasmodics, appetite-stimulating, and sleeping inductors [12]; THC, a phytocannabinoid partial CB_1_ agonist, as well as WIN55,212–2, a synthetic CB_1_ agonist, have both been proved to have an anticonvulsant effect in vitro [13] and in rodent models [14] of epilepsy and status epilepticus, being more effective than conventional antiepileptics like phenytoin and phenobarbital [15]. Furthermore, increased levels of anandamide (AEA), an endocannabinoid, have been found in cerebrospinal fluid of dogs suffering from idiopathic epilepsy compared to healthy dogs [16]. Several companies started to sell medical marihuana to be used in pets to treat chronic pain, seizures, inflammation, cancer, diabetes, nausea, anxiety and obesity. There is, however, a lack of reliable research to back those claims regarding the specific distribution of cannabinoid receptors and their associated function according to their presence in different anatomical localization within the healthy nervous system and under pathological conditions.

The expression of CB_1_ has been described in brain sections of humans using autoradiography [3, 17] and in rhesus monkeys by positron emission tomography (PET) [18]. Immunohistochemistry allows the identification of particular neuronal cells and fibres that express cannabinoid receptors because of its greater resolution [19]. Consequently, CB_1_ distribution has been extensively mapped in the mouse [20], rat [19, 21] and macaque monkey [22] CNS. In addition, CB_1_ expression has also been described particularly in the dorsal horn in rats [19, 23] and in the spinal cord of humans [17]. In the species dog, CB_1_ receptors were detected in salivary glands [24], hair follicles [25], skin and hippocampus [26]. However, a detailed analysis of the distribution of CB_1_ receptors in the CNS and PNS has not been reported in canines so far. It is well established that many conditions in dogs share striking similarities with their human counterparts thus representing suitable translational models for studying human neurological diseases including epilepsy [27], neuropathic pain [28], spinal cord injury [29] and multiple sclerosis, as described in the canine distemper virus (CDV)-induced demyelination model [30]. Thus, the species dog might help to overcome the gap between highly homogenous and standardized rodent models and clinically relevant conditions in humans. Precise knowledge of the distribution of CB_1_ within the canine nervous system are therefore of great relevance to design therapeutic strategies to manipulate the effects of the endocannabinoid system [9].

In the current study, we analyzed the spatial distribution of CB_1_ receptors in the healthy CNS and PNS of dogs from different ages. This is the first study which characterizes in detail the presence of those receptors under normal circumstances, therewith providing novel insights into the localization of CB_1_ receptors for further characterization under pathophysiological conditions.

## Materials and methods

### Animals and tissue samples

Following routine necropsy, brain and peripheral nerve samples of dogs without clinical or pathological evidence of neurologic or infectious diseases were collected and subsequently fixed in non-buffered formalin (10%) for at least 48 hours and embedded in paraffin. Serial sections (3 μm thick) were mounted on SuperFrost-Plus slides (Menzel Gläser, Braunschweig, Germany), and stained with hematoxylin and eosin (HE), a complete histological examination was performed in order to confirm the absence of histopathological lesions. Afterwards, the slides where further processed for immunohistochemistry and double immunofluorescence. A total of five dogs of different ages were included, one female and two male six-month-old Beagle dogs, one ten-year-old female Cocker Spaniel and one four-week-old female Leonberger. Tissue samples of the dogs used in this study were included in a previous study [31]. German Animal Welfare Act with the law of animal welfare, Germany (permission number: 33.9-42502-05-13A346), and the ethical guidelines of the University of Veterinary Medicine Hannover were followed for the euthanasia of the dogs. No animals were euthanized for this particular study; samples obtained and previously used in other studies were taken. The study was approved and followed the guidelines of the PhD commission of the University of Veterinary Medicine Hannover, the institutional ethics committee.

Transversal sections were cut through the brain at the level of olfactory bulb, frontal lobes, basal forebrain, thalamus, lateral and medial corpus geniculatum, hippocampus, cerebellum and brainstem. Transversal sections of the cervical, thoracic and lumbar spinal cord with their corresponding dorsal root ganglia were included, as well as a representative section of the sciatic nerve.

### Antibodies

For immunohistochemistry (IHC) and immunofluorescence (IF) a polyclonal antibody against cannabinoid receptor 1 (CB_1_, Abcam Cat# ab23703, RRID:AB_447623, 1:100 IHC, 1:15 IF), immunogen corresponding to C terminal amino acids 461–472 of Human Cannabinoid receptor 1, was included. Monoclonal antibodies included anti-glial fibrillary acidic protein (GFAP, Sigma-Aldrich Cat# G-A-5, RRID:AB_2314539, 1:300 IF), anti-2',3'-Cyclic-nucleotide 3'-phosphodiesterase (CNPase, Millipore Cat# MAB326, RRID:AB_2082608, 1:100 IF), anti-major peripheral myelin protein (P0, clone P07, 1:400 IF, Archelos et al., 1993) and anti-neurotrophin receptor p75 (p75^NTR^, American Type Culture Collection (ATCC) Cat# hb-8737, RRID:AB_2152662, 1:2 IF).

### Immunohistochemistry

CB_1_ immunohistochemistry (IHC) was performed by using the avidin-biotin-peroxidase complex (ABC) method as previously described [31, 32]. Briefly, 3 μm thick sections were dewaxed and rehydrated through a graded series of alcohols, and treated with 0.5% H_2_O_2_ to block endogenous peroxidase. Antigenic retrieval was preformed using sodium-citrate buffer (pH 6.0–6.5) for 20 minutes in the microwave at 800w. Following incubation with 20% goat serum, sections were incubated with the CB_1_ antibody overnight at 4°C. As negative control, the primary antibody was substituted with rabbit serum (1:3000; R4505; Sigma Aldrich, Taufkirchen, Germany), using the same gamma-globulin concentration as in the primary antibody formulation. Biotinylated goat-anti-rabbit IgG (1:200; BA-1000; Vector Laboratories, Burlingame, CA, USA), was used as secondary antibody and incubated for 45 minutes at room temperature, followed by incubation with ABC (VECTASTAIN-ABC Kit Standard, PK 6100, Vector Laboratories, Burlinghame, California, USA). Color development was done with 3.3’-diaminobenzidine tetrahydrochloride (0.05% solution, DAB, Sigma Aldrich, Taufkirchen, Germany) with H_2_O_2_ (0.03%, pH 7.2) for 5 min followed by slight counterstaining with Mayer’s hemalaun. Sections of tissue samples were independently examined via light microscopy (BX51, Olympus Optical CO., Tokyo, Japan). Representative images were acquired by use of photodocumentation software (DP72, Olympus Optical CO., Tokyo, Japan).

### Double immunofluorescence staining

Double immunofluorescence staining was performed on representative tissue sections as previously described [32] on 3 μm thick paraffin-embedded sections to demonstrate a possible co-localization of CB_1_ with GFAP and CNPase in the CNS, and P0 and p75^NTR^ in the PNS. Briefly, sections were simultaneously incubated with the respective primary antibodies for 90 min. Cy3-labeled goat anti-mouse (red, 1:200, Alexa Fluor 555 dye, Life Technologies) and Cy2-labeled goat anti-rabbit (green, 1:200, Alexa Fluor 488 dye, Life Technologies) secondary antibodies were used to visualize the respective antigens. Nuclear counterstaining was performed with 0.01% bisbenzimide (H33258, Sigma Aldrich, Taufkirchen, Germany) and sections were mounted with Dako Fluorescent Mounting medium (DakoCytomation, Hamburg, Germany). Antigenic expression was visualized using an inverted fluorescence microscope (BZ-9000E, Keyence GmbH, Neu-Isenburg, Germany) and examined through the BZ-II Analyzer software. All images were acquired with the same microscope settings under which control sections showed no signal. Images were transferred to Adobe Photoshop (San Jose, CA) for cropping, and they were adjusted to optimize contrast and brightness.

## Results

**Fig 1:**
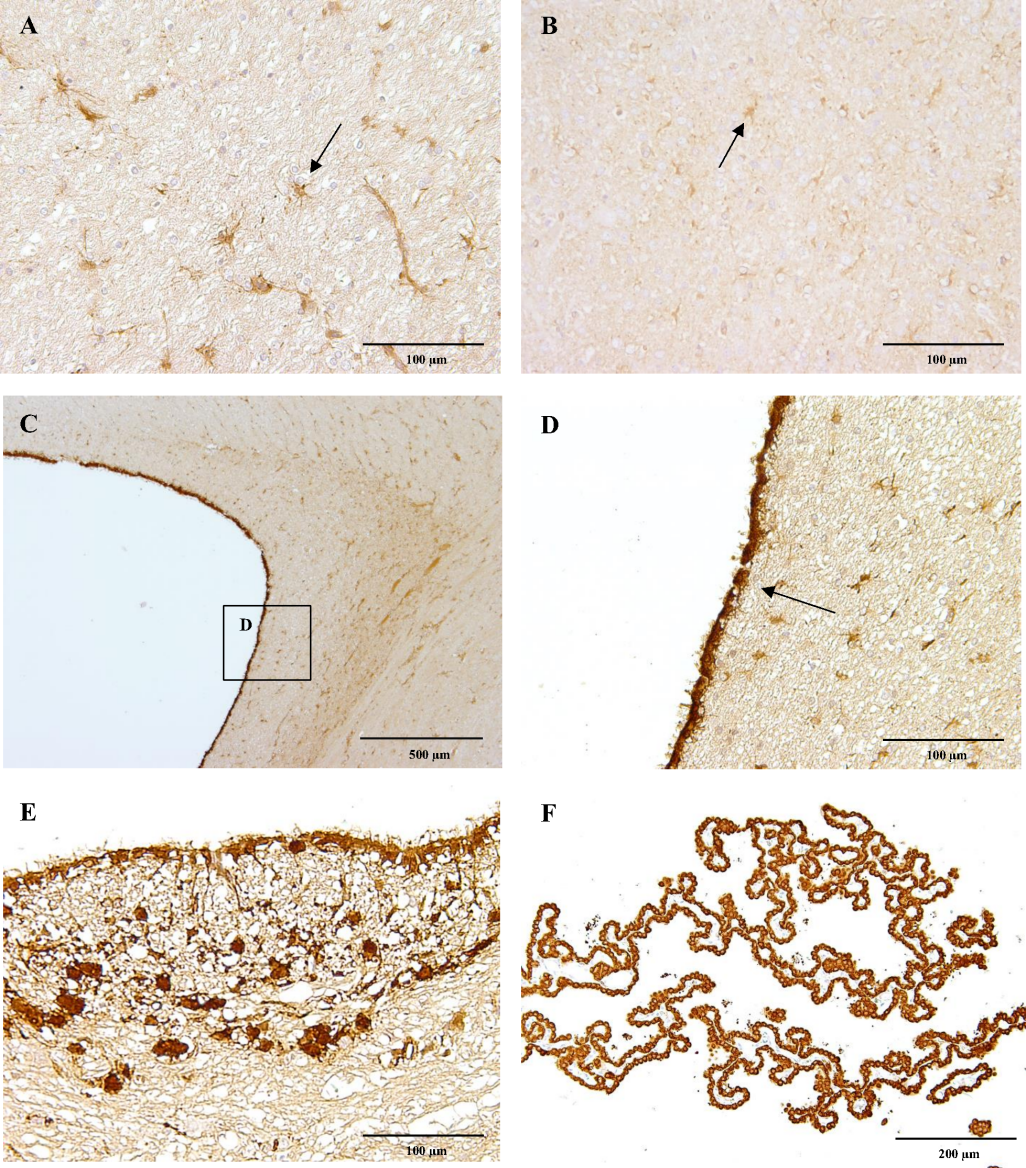
CB_1_ immunoreactivity. Astrocytes (arrow) of the cerebral white matter of a six-month-old Beagle dog showing strong CB_1_ receptor immunoreactivity (A) comparing to astrocytes of a four-week-old dog, which are only slightly positive (B). The ependymal cells (arrow) of a six-month-old dog lining the lateral ventricle strongly express CB_1_ receptor (C, D). Similarly, ependymal cells lining the fourth ventricle and scattered neuroglial cells (E) are CB_1_ receptor positive, as well as cells of the choroid plexus (F). IHC was performed using the avidin-biotin-peroxidase complex (ABC) method.

The distribution of CB_1_ immunoreactivity in anatomically related regions is described below in detail. Importantly, there were few differences in the expression of CB_1_ regarding the analysed anatomical localisations in different aged dogs. Generally, strong cytoplasmic CB_1_ immunoreactivity was observed in astrocytes both in the white and in the grey matter along the cerebrum (Fig 1A), cerebellum and spinal cord in all dogs, except in the four-week-old dog, in which only scattered astrocytes were slightly positive (Fig 1B). In addition, the cytoplasm of ependymal cells lining the lateral (Fig 1C and 1D), third, fourth (Fig 1E) ventricles and the central canal of the spinal cord; as well the choroid plexus ependymal cells (Fig 1F) strongly expressed CB_1_. Strikingly, the cytoplasm of small numbers of neuroglial cells surrounding the fourth ventricle (Fig 1E) and the central canal of the spinal cord were intensely CB_1_ positive. Within the meninges, flattened fibroblast-like cells mostly in the dura mater showed slight cytoplasmic CB_1_ immunoreactivity.

### Olfactory bulb

**Fig 2:**
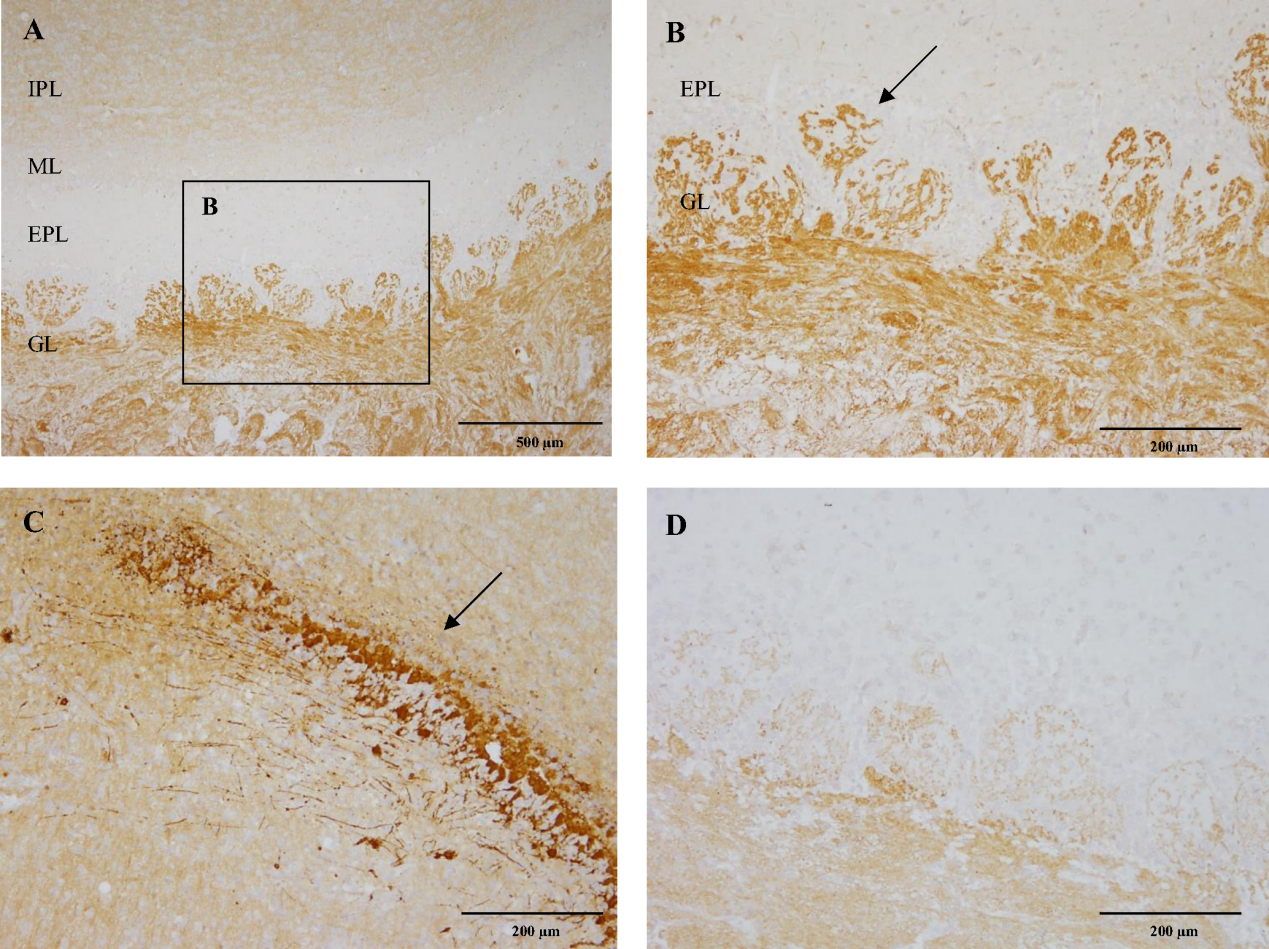
CB_1_ immunoreactivity of the Olfactory bulb. CB_1_ immunoreactivity of a six-month-old Beagle dog (A, B, C) and four-week-old dog (D). Strong immunoreactivity of the glomerular layer (GL), lack of immunoreactivity in the external plexiform layer (EPL) and mitral cell layer (ML), while moderate immunoreactivity of the internal plexiform layer (IPL) are observed in the six-month-old Beagle dog (A). Detailed immunoreactivity of the GL (arrow) is depicted in B. In the six-month-old Beagle dog, a population of cells within the internal granule cell layer (arrow) is strongly CB_1_ receptor positive (C). Contrary, the glomerular layer in the four-week-old dog was only slightly CB_1_ receptor positive (D). IHC was performed using the avidin-biotin-peroxidase complex (ABC) method.

In the main olfactory bulb, network of fibres were intensely stained with CB_1_ in the glomerular layer (GL) (Fig 2A). CB_1_ immunoreactivity occurred in a network of fibres that surrounded unstained neuronal soma (Fig 2B). Immunoreactivity was also found in the fibres of the internal plexiform layer (IPL) (Fig 2A). In addition, a population of cells within the internal granule cell layer were strongly CB_1_ positive (Fig 2C). No immunoreactivity was observed in the external plexiform layer (EPL) or in the mitral cell layer (ML). However, mitral cells axons were moderately CB_1_ positive. In the four-week-old dog, only the glomerular layer expressed moderate CB_1_ immunoreactivity and all other layers were devoid of immunostaining (Fig 2D).

### Cerebral cortex (neocortex-frontal lobe)

**Fig 3:**
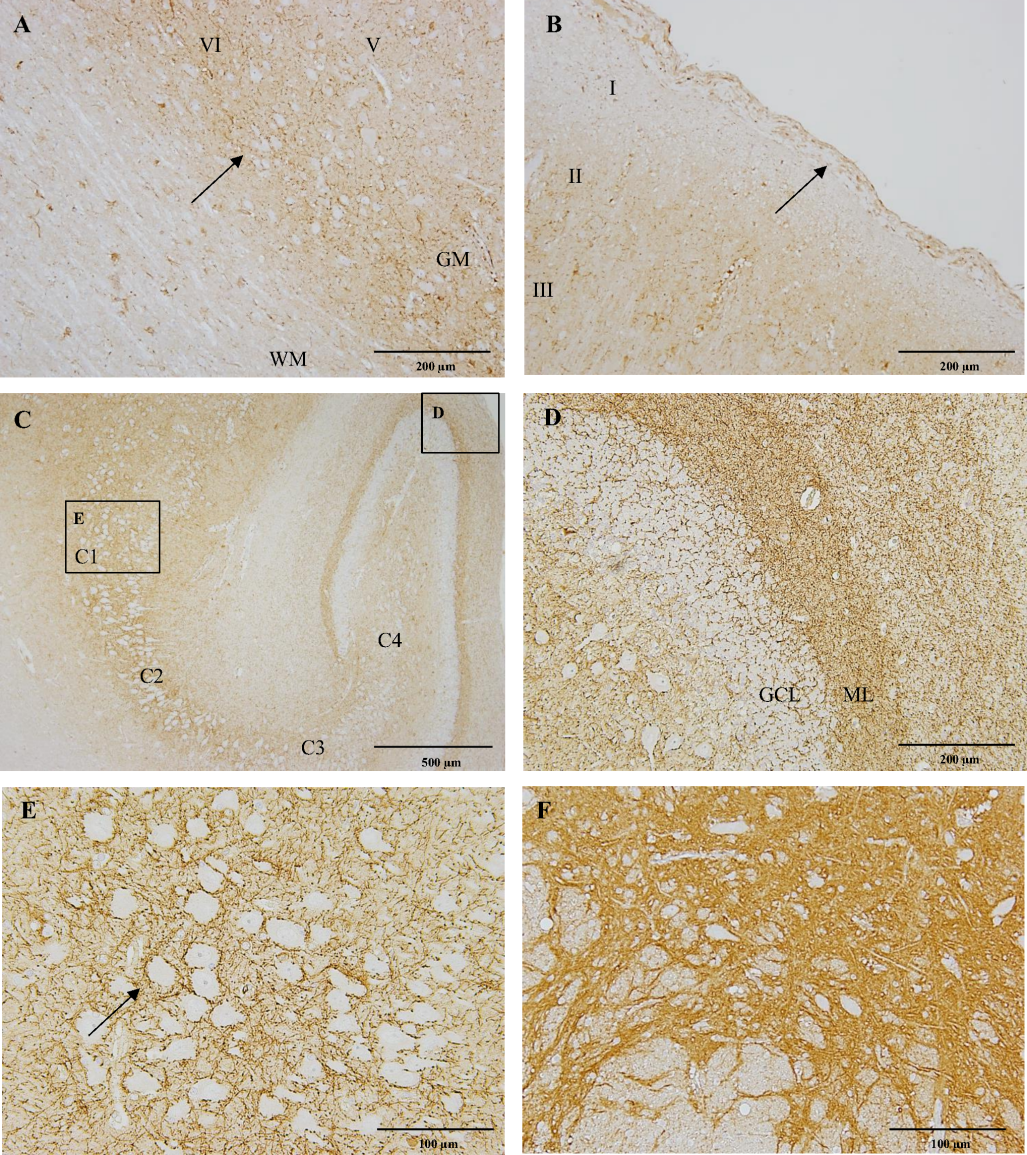
CB_1_ immunostaining of the cerebral cortex, hippocampus and substantia nigra in a six-month-old Beagle dog. Within the frontal lobe of the cerebral cortex, there is an intense CB_1_ immunoreactivity of fibres surrounding unstained neuronal bodies in layers V and VI (A; arrow). In figure B, layer I appears almost devoid of CB_1_ immunoreactivity and layer II and III express strong immunoreactivity. Notice that the meninges show positive flattened fibroblast-like cells in the dura matter (B, arrow). The hippocampus shows progressive decrease in the immunoreactivity from C1 to C4 (C). In figure D, the dentate gyrus of the hippocampus depicting strong dot-like CB_1_ immunoreactivity in the molecular layer (ML). Interestingly, the granule cell layer (GCL) appears devoid of CB_1_ immunoreactivity. The stratum pyramidale shows strong CB_1_ immunoreactive fibres surrounding unstained pyramidal neuronal bodies in the C1 (E; arrow). In figure F, strong CB_1_ immunoreactivity is observed in fibres of the substantia nigra pars reticulata. IHC was performed using the avidin-biotin-peroxidase complex (ABC) method. WM: white matter; GM: grey matter.

The grey matter of the neocortex expressed strong CB_1_ immunoreactivity in the external granular layer (II), external pyramidal layer (III), inner granular layer (IV), inner pyramidal layer (V) and multiform layer (VI) (Fig 3A and 3B). This intense immunoreactivity of the fibres was presented in a dot-like pattern surrounding the unstained neuronal bodies (Fig 3A). The densest expression was found in the II, III, V and VI layers of the frontal lobe while the molecular layer (I) appeared almost devoid of CB_1_ immunostaining (Fig 3B). In the four-week-old dog and the ten-year-old dog, the intensity of the immunoreactivity was lower comparing to the other dogs.

### Hippocampus

Within the hippocampus, strong dot-like CB_1_ immunostaining was associated with a dense network of fibres in the stratum pyramidale surrounding the unstained pyramidal neuronal bodies (Fig 3C and 3E). A progressive decrease in the immunoreactivity from CA1 to CA4 was seen (Fig 3C). In the hippocampal polymorphic layer and the molecular layer, the fibres were less intensely stained. In the dentate gyrus, CB_1_ immunoreactivity was associated with fibres in the molecular layer with the most intense staining occurring adjacent to the granule cell layer (Fig 3D). The granule cell layer lacked CB_1_ expression (Fig 3D).

### Basal ganglia and lateral and medial geniculate nucleus

Intense CB_1_ immunoreactive fibres were observed in the globus pallidus of the basal nuclei. The expression of CB_1_ was observed only in scattered fibres in the lateral and medial geniculate nucleus.

### Midbrain

Strong CB_1_ immunoreactivity was observed in the fibres surrounding unstained neuronal bodies in the substantia nigra, denser towards pars reticulata (Fig 3F). There were intensely stained fibres from all directions at the level of the oculomotor nucleus and red nucleus. In addition, moderate CB_1_ immunoreactivity was observed in the fibres of the periaqueductal gray (PAG) and in the soma of neurons.

### Cerebellum

**Fig 4:**
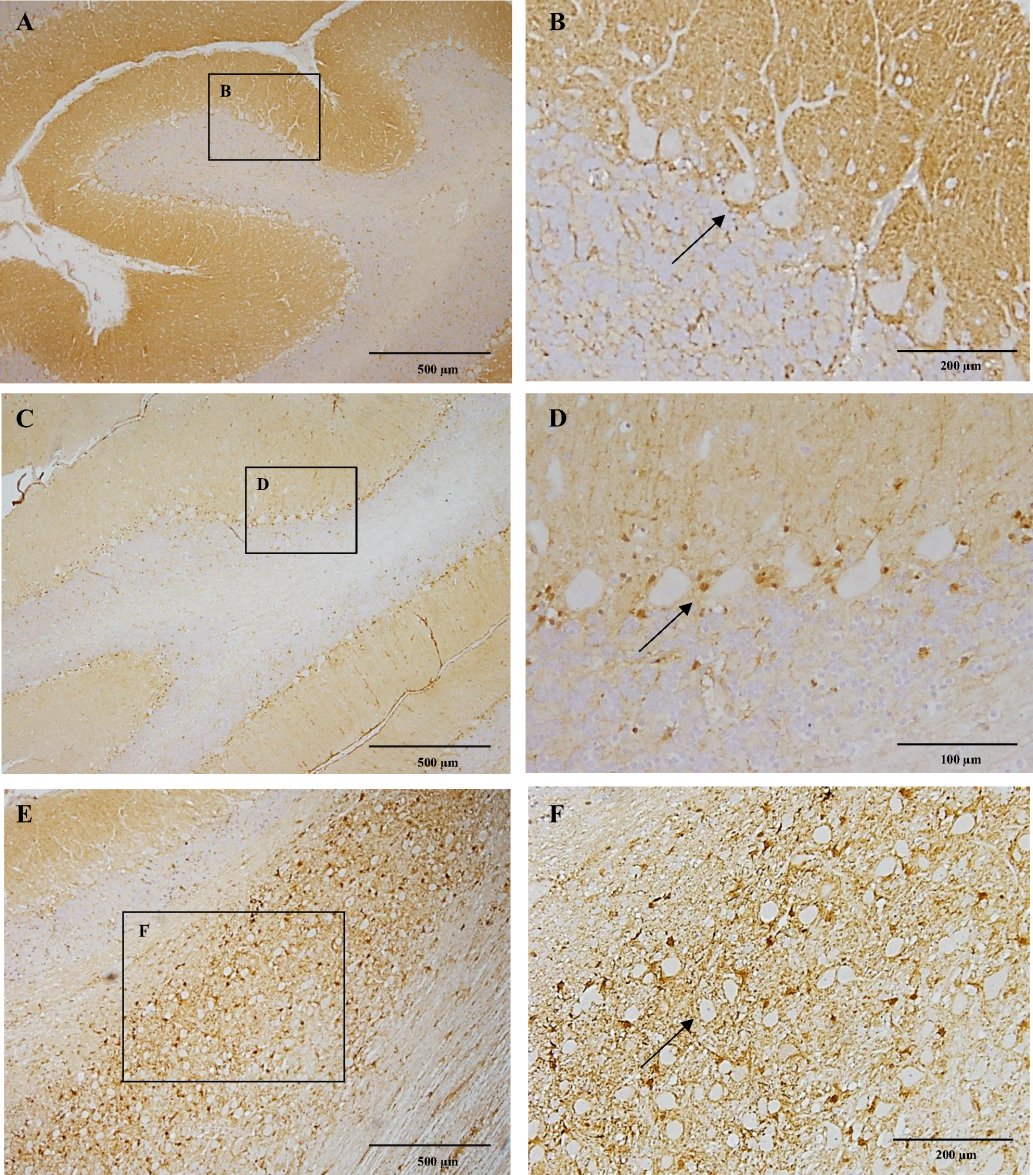
CB_1_ immunoreactivity of the cerebellum and choclear nuclei. In figure A notice strong CB_1_ immunoreactivity within the molecular layer of the cerebellar cortex in a six-month-old Beagle dog. Figure B depicting in detail immunonegative Purkinje cells surrounded by strong immunorreactive fibers particularly in the basal portion (arrow). In the ten-year-old dog, there is a slight immunoreactivity in the molecular layer of the cerebellar cortex (C). Purkinje cells surrounded by a dot-like immunoreactivity appear devoid of immunoreactivity in the ten-year-old dog (D; arrow). The cochlear nucleus in a six-month-old dog showing strong CB_1_ immunoreactivity (E). In figure F detail of the cochlear nucleus with strong CB_1_ immunoreactivity surrounding the unstained neuronal bodies (arrow). IHC was performed using the avidin-biotin-peroxidase complex (ABC) method.

Strong CB_1_ immunoreactivity was observed homogenously within the molecular layer of the cerebellar cortex (Fig 4A and 4B). Small numbers of Purkinje cells showed slight cytoplasmic immunoreactivity. Interestingly, strong immunoreactivity was present surrounding the Purkinje cells bodies, particularly in the basal portion of the cells (Fig 4B). The underlying granule cell layer remained negative, with just few scattered positive fibres surrounding unstained cellular bodies (Fig 4B). In the ten-year-old dog the staining pattern remained alike, however, the molecular layer showed moderate to slight CB_1_ immunoreactivity, while other layers remained negative (Fig 4C and 4D), Purkinje cells were surrounded by dot immunoreactivity (Fig 4D).

### Medulla oblongata

Surrounding the neuronal bodies of the cochlear nucleus (Fig 4E) and the nucleus of the spinal tract of the trigeminus, a strong dot-like CB_1_ immunoreactivity was observed while the neuronal cytoplasm were completely negative (Fig 4F).

### Spinal cord

**Fig 5:**
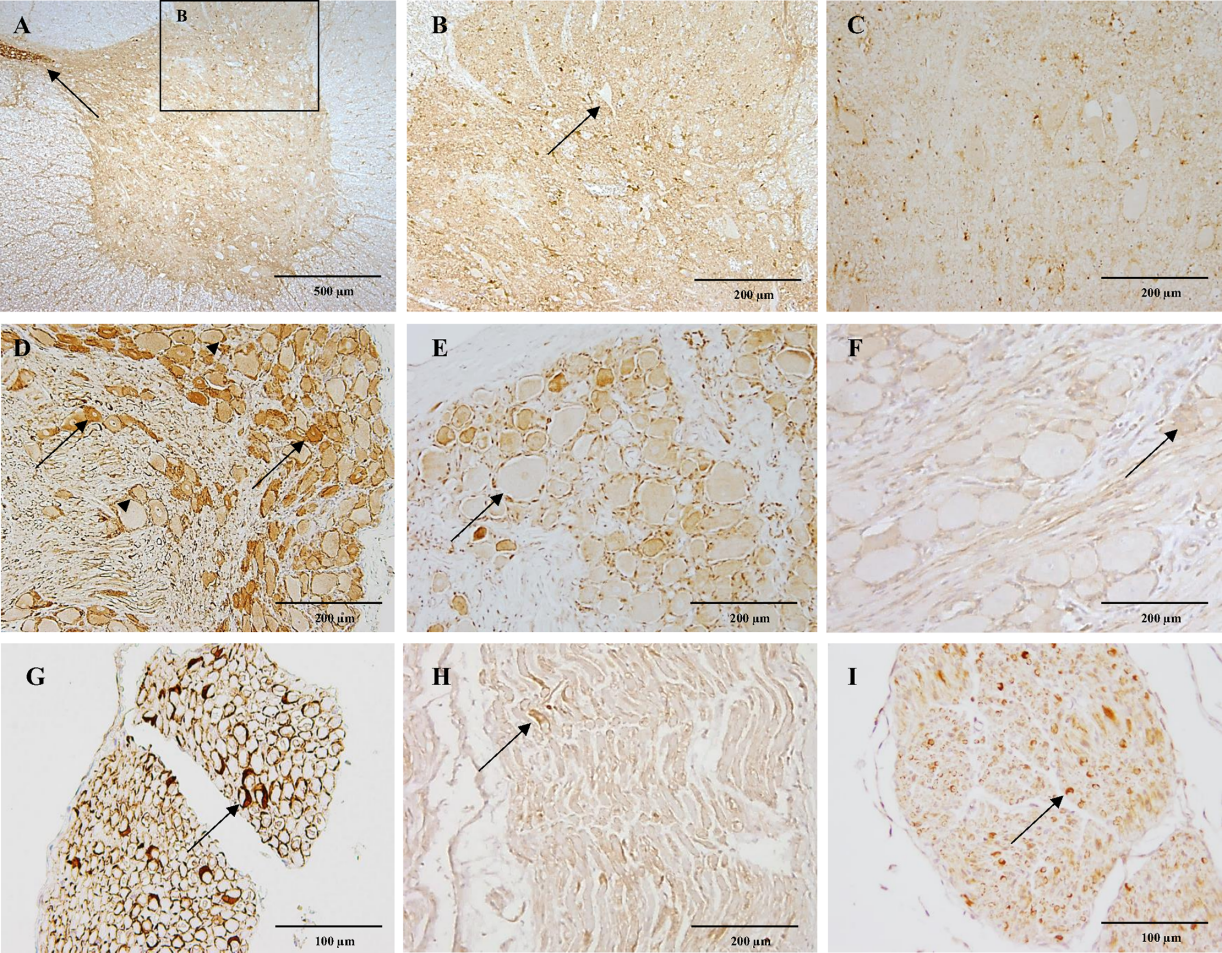
CB_1_ immunoreactivity in the spinal cord, dorsal root ganglia and peripheral nerve. In figure A, strong CB_1_ immunoreactivity is shown in the grey matter of the cervical spinal cord of a six-month-old dog and the cytoplasm of ependymal cells lining the central canal (A; arrow). Within the dorsal horn, CB_1_ immunoreactivity appears surrounding unstained neuronal bodies (B; arrow). In the cervical spinal cord of a ten-year-old dog notice slight immunoreactivity of the grey matter (C). Figure D showing the cervical dorsal root ganglia of a six-month-old dog with slight immunoreactivity of large neurons and strong CB_1_ immunoreactivity of small dark neurons (arrows) and satellite cells (arrowheads). The thoracic dorsal root ganglia of a ten-year-old dog with moderate CB_1_ immunoreactivity of small dark neurons and satellite cells, large neurons show slight immunoreactivity (E; arrow). The cervical dorsal root ganglia of a four-week-old dog depicting scattered large and small neurons and satellite cells with slight CB_1_ immunoreactivity (F; arrow). In figure G the cervical spinal nerve of a six-month-old dog shows strong CB_1_ expression in Schwann cells ensheating axons (arrow). Few Schwann cells show moderate CB_1_ immunoreactivity (arrow) in a thoracic spinal nerve of a ten-year-old dog (H). The cervical spinal nerve in the four-week-old dog shows moderate CB_1_ immunoreactivity of scattered Schwann cells (I; arrow). IHC was performed using the avidin-biotin-peroxidase complex (ABC) method.

Within the grey matter of the cervical, thoracic and lumbar spinal cord, strong CB_1_ immunoreactive fibres were observed in the dorsal horn, intermediate region and ventral horn (Fig 5A). CB_1_ dot-like immunostaining was present surrounding the neuronal bodies (Fig 5B). In addition very few neurons showed slight cytoplasmic immunoreactivity within the ventral and dorsal horns (Fig 5B). In the four-week-old and the ten-year-old dogs, the intensity of the immunoreactivity in the grey matter was lower comparing to the six-month-old dogs (Fig 5C).

### Dorsal root ganglia

Within the dorsal root ganglia (DRG) of the cervical, thoracic and lumbar spinal cord segments large neurons showed slight cytoplasmic CB_1_ immunoreactivity, while small dark neurons strongly expressed CB_1_ (Fig 5D). In addition, satellite cells were strongly immunopositive (Fig 5D). In the ten-year-old dog the immunostaining pattern remained, nevertheless, the overall DRG immunostaining was weaker (Fig 5E). In the four-week-old, however, only scattered large and small dark neurons and satellite cells were slightly positive (Fig 5F).

### Peripheral nerve

CB_1_ immunostaining within the thoracic spinal nerve revealed strong expression in randomly distributed Schwann cells ensheathing axons (Fig 5G). In the four-week-old and the ten-year-old dogs, the intensity of the CB_1_ immunoreactivity was lower in positive Schwann cells (Fig 5H and 5I). Moreover, in the ten-year-old dog only few Schwann cells showed a moderate positive immunoreactivity (Fig 5H).

### Double immunofluorescence tracking of glial cells expressing CB_1_ receptors in the CNS and PNS

**Fig 6:**
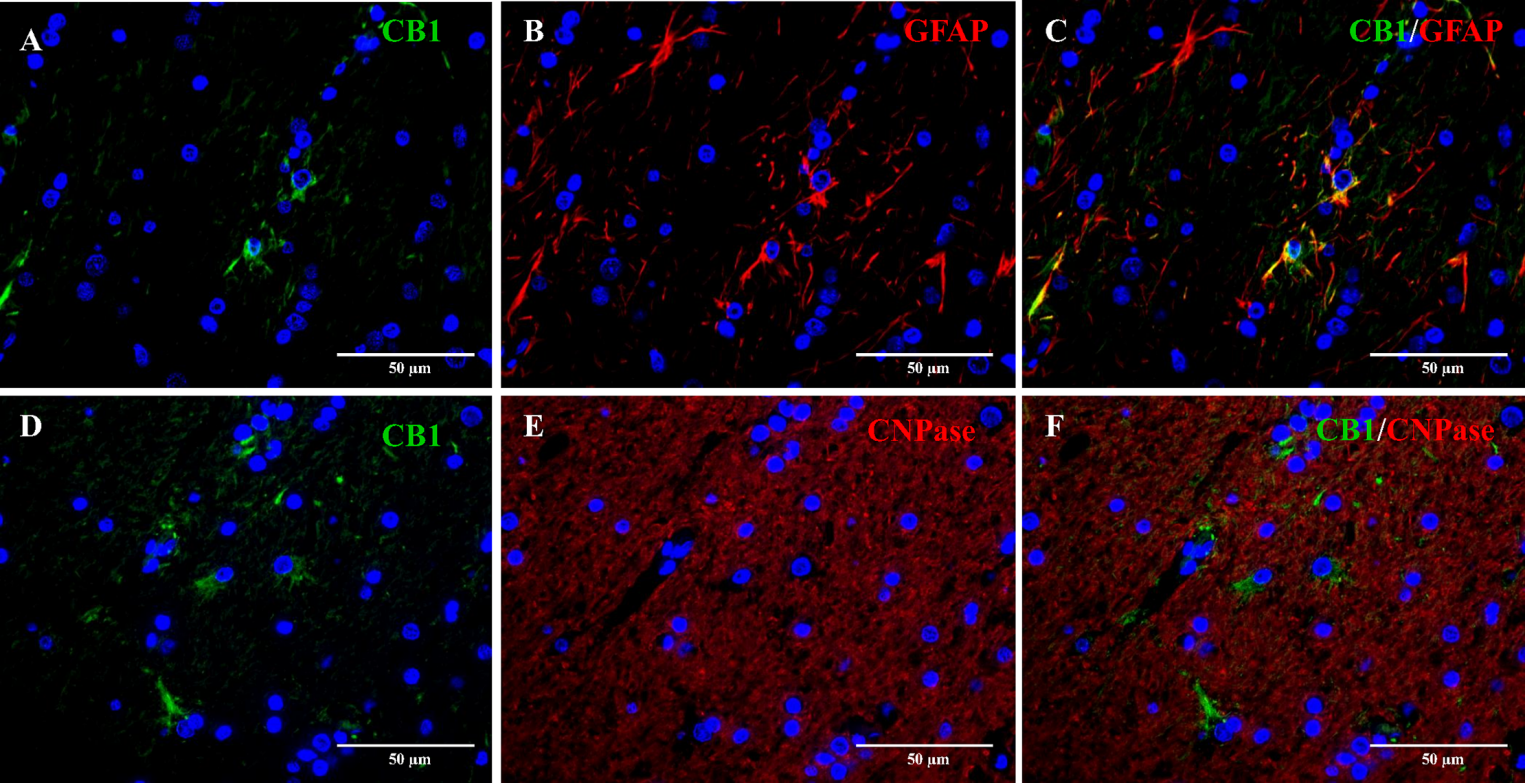
Double immunofluorescence staining of the cerebral white matter of a six-month-old Beagle dog. Double immunofluorescence staining of CB_1_ (green, A) with GFAP (red, B) reveals co-localization in about 20% astrocytes (C). CNPase expression (red, E) and CB_1_ (green, D) do not co-localize, suggesting a lack of expression of CB_1_ receptors by mature oligodendrocytes (F). Nuclear staining (blue) with bisbenzimide.

In order to specifically identify the glial cell types expressing CB_1_ receptors in the CNS and PNS, double immunofluorescence was performed in a representative case of a six-month-old beagle dog. Co-localization of CB_1_ with the astrocytic marker GFAP was observed in about 20% of the GFAP-positive astrocytes, indicating that only a subpopulation of astrocytes does express CB_1_ receptors (Fig 6A and 6C). On the other hand, no co-expression was present among CB_1_ and the mature oligodendrocytic marker CNPase (Fig 6D and 6F).

**Fig 7:**
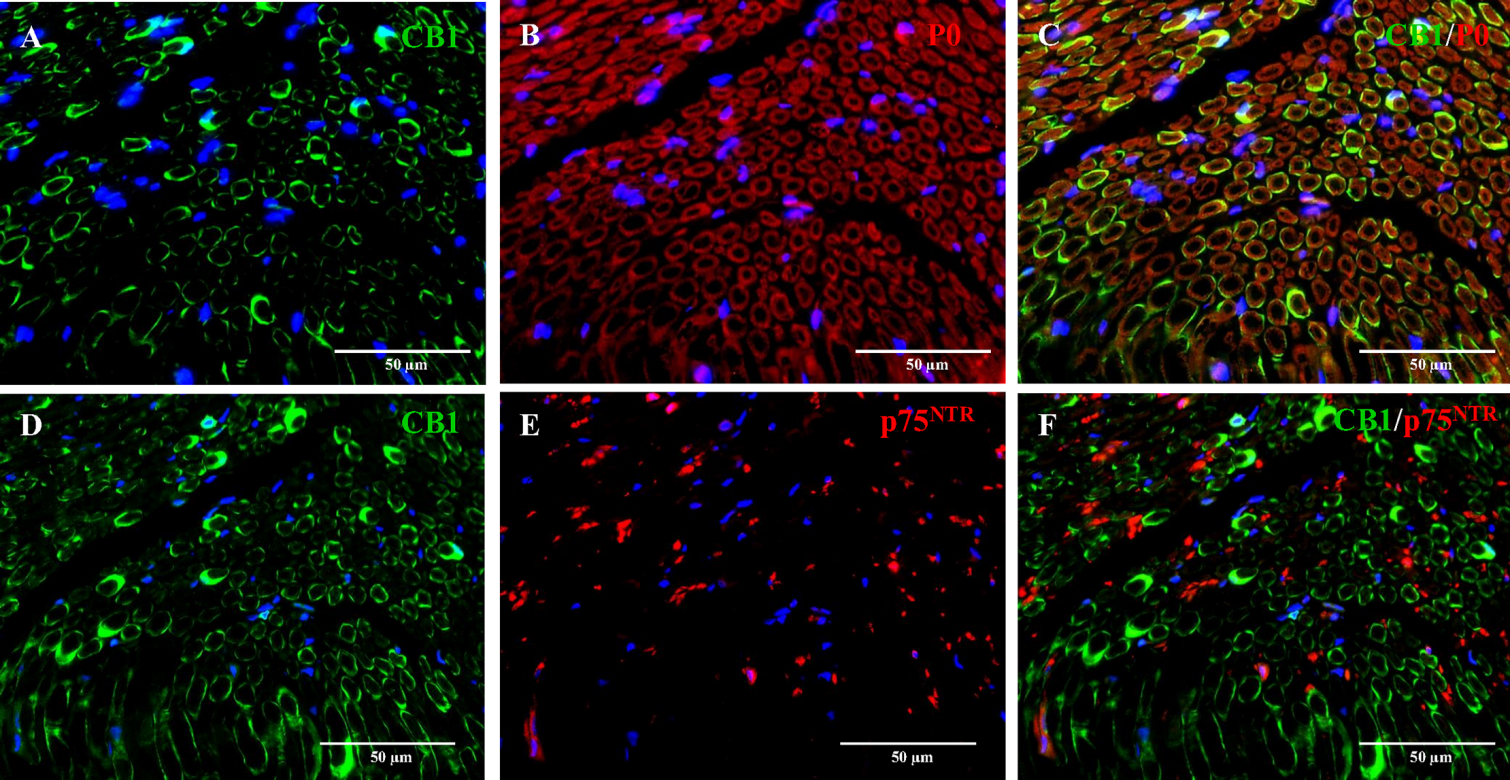
Double immunofluorescence staining of the sciatic nerve of a six-month-old dog. P0, a marker for myelinating Schwann cells (red, B) and CB_1_ (green, A) co-localize in about 100% of Schwann cells (C). p75^NTR^ (red, E) and CB_1_ (green, D) do not co-localize (F), suggesting the absence of CB_1_ receptors in non-myelinating Schwann cells. Nuclear staining (blue) with bisbenzimid.

Interestingly, double immunolabelling of the sciatic nerve showed co-localization of CB_1_ and P0, a marker for myelinating Schwan cells, in about 100% of the Schwann cells (Fig 7A and 7C). On the contrary, no co-expression was found among CB_1_ and the non-myelinating Schwann cells marker p75^NTR^ (Fig 7D and 7F).

## Discussion

This study describes the first detailed spatial distribution of CB_1_ receptors in the healthy canine CNS and PNS. A commercially available antibody against human CB_1_ was used, corresponding to C terminal amino acids 461–472 of human cannabinoid receptor type 1. The CB_1_ protein sequence is highly conserved across mammalian species [33], moreover, crossreactivity of this specific CB_1_ antibody with canine tissue has been previously demonstrated in peripheral tissues, hippocampus and cerebellum of adult dogs [26, 34] and in canine embryos [35].

The distribution of CB_1_ receptors in the CNS of dogs in this study was similar but no equal to those of previews studies made in rats [21] and monkeys [22] using C-terminus antibodies, and those of mice [20], rats [19] and monkeys [22] using N-terminus antibodies.

The presence of cannabinoid receptors in the canine CNS was first reported by the autoradiographic studies of Herkenham in 1990, who found the densest binding of a radiolabeled synthetic cannabinoid in the cerebellar molecular layer, followed by the globus pallidus, substantia nigra pars reticulate, hippocampal dentate gyrus and the neocortex [3]. This pattern, with few variations remained for humans, rhesus monkeys, rats and guinea pigs [3]. Our study using immunohistochemistry shows similar results regarding the overall distribution of CB_1_ in healthy CNS of dogs.

In the current study, the distribution of CB_1_ immunostaining consisted mostly on strongly positive network of fibres in specific regions such as the olfactory bulb, cerebral cortex, cerebellar cortex, hippocampus, basal ganglia, cochlear nucleus, nucleus of the spinal tract of the trigeminus and grey matter of the spinal cord. This particular distribution might be due to the fact that the CB_1_ receptors are mainly expressed in axons and pre synaptic terminals [36–38], emphasizing the important role of this receptor as a modulator of neurotransmitter release at specific synapses [9, 39, 40]. CB_1_ receptors, however, have also been found on postsynaptic structures [22, 23, 41], glial cells [42–45] and peripheral cells such as cells of the striated ducts of the parotid and mandibular glands, keratinocytes, fibroblasts and macrophages [24, 25, 46–48].

Strikingly, ependymal cells lining the ventricular system and the central canal of the spinal cord, and a small numbers of neuroglial cells surrounding the fourth ventricle and the central canal expressed the strongest cytoplasmic CB_1_ immunoreactivity. Cells surrounding the central canal of the spinal cord are a source of stem/precursor cells that may give rise to neurons, astrocytes, or oligodendrocytes [49]. The ependymal region in the spinal cord has been shown to express CB_1_ in rodents [50] and humans [51]. Even a subpopulation of ependymal cells named “CB_1_ high cell” has been described in both species [49, 51], co-expressing stem/precursor cell markers in rats [49]. Control of proliferation of brain progenitors/stem cells through CB receptor activation has been shown *in vitro* [52, 53]. Furthermore, “CB_1_ high cells” proliferate during early postnatal development and after spinal cord injury (SCI) in adult rats, but not in the unlesioned spinal cord [49]. Aguado and others showed that endocannabinoid signalling controls neural progenitor differentiation into astroglial cells in postnatal and adult mice [52]. Nevertheless, further studies are needed to fully understand the potential of cannabinoids on neurogenesis in the dog and other species [49].

In the olfactory bulb, we demonstrated a network of fibres intensely stained with CB_1_ in the glomerular layer and moderate staining of the internal plexiform layer surrounding unstained neuronal soma. Despite lack of immunostaining of the mitral cell layer, mitral cell axons were moderately CB_1_ positive. Furthermore, a population of cells located in the internal granule cell layer expressed strong cytoplasmic CB_1_ immunoreactivity. In mice [54] and rats [19, 21], the strongest CB_1_ immunoreactivity has been detected in the fibres of the inner granule cell layer, followed by the inner plexiform layer, however, only surrounding unstained cell bodies. Soria-Gómez and others demonstrated that the endocannabinoid system controls food intake via olfactory processes in mice [54].

CB_1_ receptors are described to be densely expressed in all regions of the cortex in mice, rats, monkeys and humans [17, 19, 22, 55]. While the general laminar pattern of CB_1_ immunoreactivity between species seems preserved [9], the densest expression appears to be in the III and V in primates [9] and within layers II, III and VI in mice [21] and rats [19], while layer I appears almost devoid of CB_1_ receptors in these species [9, 21]. We found intense CB_1_ immunoreactivity presented in a dot-like pattern of the fibres surrounding unstained neuronal bodies, as previously described [21], within layers II, III, IV, V, and VI of the neocortex, with the densest expression found in the II, III, IV and VI layers of the frontal lobe. In humans and monkeys, the laminar pattern has been widely studied and shows distinctive laminar density across the different regions, showing higher CB_1_ expression in the prefrontal cortex [22]. In the monkey, neocortex immunoreactivity is primarily contained in cells and axon terminals that show morphological features of GABAergic neurons [22]. Moreover, the majority of high CB_1_ expressing cells in the rat forebrain are GABAergic neurons [56].

The strongest immunoreactivity we found in the hippocampus was associated with fibres within the molecular layer of the dentate gyrus with the most intense staining occurring adjacent to the granule cell layer, which lacked CB_1_ expression. A similar pattern has been found in macaques [22], mice [20] and rats [19], where the highest CB_1_ density was found within the molecular layer of the dentate gyrus, while the granule cell layer appeared to be completely devoid of CB_1_ immunoreactivity. In immunohistochemical studies made in rats [19], mice [20, 57] and macaques [22] strong immunoreactivity occurred in the *Cornu Ammonis* (CA) regions of the hippocampus within the pyramidal layer. Interestingly, the cell bodies of pyramidal neurons in CA1-CA3 fields appeared to be unstained but surrounded by a dense plexus of highly immunoreactive fibres [19–22, 57]. Indeed, we found strong CB_1_ immunostaining associated with a dense network of fibres in the stratum pyramidale also surrounding immunonegative pyramidal neuronal bodies. Specific expression of CB_1_ receptor has been already reported in the hippocampus of healthy dogs. Dot-like structures with CB_1_ immunoreactivity were found lining the external surface of neuronal cell bodies in the 4 regions of the CA where the cytoplasm of neurons did not have CB_1_ immunoreactivity [26]. Our findings agree with this previous study. Interestingly, we found a progressive decrease in the immunoreactivity from CA1 to CA4. Campora and others described a similar pattern in the canine CA [26]. In rats [58] and humans [37] most of CB_1_ immunoreactive neurons in the hippocampus are GABAergic, and are involved in mechanism by which cannabinoids impair memory and associational processes [37]. Expression of CB_1_ is markedly increased specially in the stratum pyramidale (CA1-CA3) and molecular layer of the dentate gyrus in different mouse models of epilepsy [57, 59, 60] and human patients with epilepsy [60]. This CB_1_ upregulation may be a compensatory mechanism of excitatory neurons to strengthen the negative feedback loop of the endocannabinoid system and to down-regulate neurotransmitter release [57].

The subcortical nuclei with the highest level of CB_1_ receptor expression are the basal ganglia, including the globus pallidus and substantia nigra pars reticulate in rats [19, 21], rhesus monkeys [3] and humans [3, 17]; and account for the complex effects of cannabinoids on motor behavior [61–63]. In dogs, however, the expression is lower compared to these species [3]. We found intense CB_1_ immunoreactive fibres in the globus pallidus and in the fibres surrounding unstained neuronal bodies in the substantia nigra; this immunoreactivity was stronger towards the pars reticulata.

Our results show moderate CB_1_ immunoreactivity in fibres and soma of neurons of the periaqueductal grey (PAG). CB_1_ immunoreactivity has been found on cell bodies [64] as well as axons and dendrites of the PAG in healthy rats [19, 64]. Furthermore, expression of CB_1_ immunoreactive neurons is increased after immobilization stress [64]. Stress activates neural systems that inhibit pain sensation depending on neural pathways projecting from cortical neurons to the PAG and descending to the brainstem and spinal cord suppressing nociception [64]. Moreover, antinociceptive effects of cannabinoids in the PAG have been proven in rats [65].

Strong homogeneous staining has been described in the molecular layer of the cerebellar cortex and surrounding the immunonegative Purkinje cells bodies in rats [19], mice [20] and macaques [22], particularly in their basal areas, corresponding to initial axonal segments [20] or basket cell processes [21, 22]. We found identical patterns of immunoreactivity in this particular region. Higher receptor-binding levels have been found in the canine cerebellum compared to humans [3], which might induce less motor depression in humans under effects of THC [66, 67]. Interestingly, the use of THC and cannabinoid analogs in experimental studies showed ataxia and even prostration at higher dosages in dogs [67, 68]. High concentrations of cannabinoid expression in the basal ganglia and cerebellum are consistent with their involvement in the initiation and coordination of movement [3, 69] and explain this behavioural changes in dogs at high doses of THC and cannabinoid analogs.

At the level of the medulla oblongata a strong dot-like CB_1_ immunoreactivity was observed only surrounding the neuronal bodies of the cochlear nucleus and the nucleus of the spinal tract of the trigeminus. Such beaded fibers were described in the spinal trigeminal tract and spinal trigeminal nucleus in rats [19].

Within the grey matter of all spinal cord sections, strong CB_1_ immunoreactive fibres were observed in the dorsal horn, intermediate region and ventral horn. CB_1_ dot-like immunostaining was present surrounding the body of groups of neurons. Few neurons showed slight cytoplasmic immunoreactivity within the dorsal and ventral horns. In humans, strong CB_1_ immunoreactivity has been found in dorsal horn, lamina X and ventral horn [51]. Immunoreactive cell bodies have been found in the lamina X in rats [23, 70] and according to one study through all grey matter [71].

DRG larger cells seamed devoid of immunoreactivity in rats [72]. Interestingly, cultured rat DRG [73] and *in-situ* hybridization studies [74] showed that most CB_1_ immunoreactive neurons are small cells. According to our results, large neurons showed slight cytoplasmic expression and small dark neurons expressed high CB_1_ immunoreactivity. Indeed, satellite cells strongly expressed CB_1_. The presence of CB_1_ receptors in the DRG and the dorsal horn may explain some analgesic effects of cannabinoids [71]. Cannabinoids have been widely reported to produce antinociception in several animal models [75–78] and the effects are mediated through CB_1_ receptors through peripheral [78], spinal [79] and supraspinal [76] mechanisms.

Moderate to strong cytoplasmic CB_1_ immunoreactivity was observed in astrocytes both in the white and grey matter along the cerebrum, cerebellum and spinal cord in all dogs. However, co-expression of CB_1_ with the astrocytic marker GFAP was observed only in about ~20% of astrocytes in the cerebral white matter. CB_1_ expression has been evidenced *in-situ* in the cytoplasm and processes of astrocytes in rats [43, 72, 80]. *In vitro* studies suggest that cannabinoids may influence astrocyte function [81]. Bidirectional neuron-astrocyte communication has been demonstrated [82–84]. Furthermore, astrocytes have been shown to be activated by endocannabinoids released by neurons [42]. Forming a “tripartite synapse” where an exchange of information with the synaptic neuronal elements occurs, responding to synaptic activity and thus regulating synaptic transmission [84]. Interestingly, increased CB_1_ expression has been shown in astrocytes of the hippocampus of epileptic rats [45]. Therefore, astrocytes should be taken into account when assessing the overall effects of cannabinoids [44] particularly in epilepsy [45].

No co-expression was found among CB_1_ and the mature oligodendrocytic marker (CNPase) in the cerebral white matter. Interestingly, previous research has shown CB_1_ expression in vitro and in vivo in oligodendrocytes of healthy rat brain and spinal cord [50, 85–87]. CB_1_ expression was evidenced in oligodendrocyte progenitor cells (OPCs) in cultures [50, 85] and in oligodendrocytes of postnatal and adult cerebral [86] and spinal cord white matter in rats [87]. In humans however, CB_1_ receptor expression has been found in OPCs and adult oligodendrocytes within multiple sclerosis (MS) plaques, but not in healthy brain tissue [88].

Strikingly, CB_1_ immunostaining within the sciatic nerve revealed strong expression in randomly distributed Schwann cells ensheathing axons. Moreover, co-expression of CB_1_ and P0, a marker for myelinating Schwan cells was found in 100% of the Schwan cells stained. CB_1_ expression in peripheral nerve fibres have been described in rats [71]. However, CB_1_ expression in Schwann cells has not been previously reported to our knowledge. Nevertheless, the presence of CB_1_ in myelinating Schwann cells might have a role in myelination processes.

Regarding the age of the dogs analysed, we found a lower general CB_1_ expression in the fourth-week-old dog. CB_1_ expression have been described in the fetal and neonatal human brain showing that the density of receptor expression was generally similar in both [89] or even higher in neonatal human brains [17]. A lower CB_1_ receptor expression has been found in aged rats in specific regions, being most prominent in the cerebellum, cerebral cortex [90], basal ganglia [91], and less prominent in the hippocampus [90]. These findings agree with our results and might be related to the decline of motor coordination and cognitive performance observed in normal ageing [92].

The small number of cases of animals with different ages does not allow us to draw definite conclusions regarding particularities among younger and older dogs. However, it is clear that the overall distribution of CB_1_ receptors is preserved in dogs at examined ages. The intensity of the expression, however, is known to change during development and aging.

## Conclusions

These results represent the first detailed spatial distribution of CB_1_ receptors in the healthy canine CNS and PNS. Our results agree with the overall distribution of CB_1_ receptors reported in other species. The high CB_1_ expression found in the cerebral and cerebellar cortex, *Cornu Ammonis* (CA) and dentate gyrus of the hippocampus, globus pallidus and substantia nigra spinal cord and DRG might relate to the effects of cannabinoids on cognition, memory, motor functions and pain sensitivity. Moreover, expression on ependymal cells and neuroglial cells relate to the effects on neurogenesis and gliogenesis modulation. Finally, CB_1_ expression on myelinating Schwann cells points out potential roles of the encocannabinoid system in myelination. Our results provide a solid basis for further studies to elucidate the physiological consequences and the implication of CB_1_ receptors in pathological conditions with the future aim to manipulate them in pharmacotherapy.
